# Signal detection in neural populations: the importance of heterogeneity

**DOI:** 10.1186/1471-2202-12-S1-P205

**Published:** 2011-07-18

**Authors:** Jorge F Mejias, André Longtin

**Affiliations:** 1 Department of Physics and Centre for Neural Dynamics, University of Ottawa, Ottawa, Ontario, K1N 6N5, Canada

## 

Neural ensembles process sensory information by employing a wide set of strategies and mechanisms. When attempting to investigate some of these mechanisms, most theoretical and computational studies assume networks of identical neurons which may work together in a collective fashion. Actual neural systems, however, display a prominent heterogeneity in neuron properties. This seems to be relevant in many contexts, such as in synchronization of inhibitory networks [1,2], global detection of weak signals [3,4], or envelope and temporal coding [5]. Still, the effect of heterogeneity in the dynamics of neural populations has not been fully understood up to now.

We present here a detailed theoretical and numerical study of the implications of heterogeneity in the properties of neural populations. We assume a population of all-to-all connected excitatory integrate-and-fire neurons, each one of them with a different distance-to-threshold value. Such heterogeneity serves to reflect some of the variability in the individual excitability properties of neurons found in actual neural systems. Our study shows important effects of heterogeneity in several quantities of interest, such as the network mean firing rate and mean coefficient of variation (CV) of the firing rate. These effects are found for values of the heterogeneity which are within the observed physiological range, which denotes the importance of considering heterogeneity in realistic modeling of neural populations. Our results also indicate a strong effect of the heterogeneity in the synchronization properties of the network.

In addition to these findings, the model reveals an interesting heterogeneity-induced phenomenon. More precisely, in the presence of heterogeneity in neural excitability, neural populations may employ two well differentiated strategies to detect and process weak incoming signals. With one of them, the network firing rate is able to faithfully reproduce the shape of the incoming signal, while according to the other strategy the signal is encoded in narrowly localized population bursts. Both strategies optimally codify the external signal for a certain value of the heterogeneity -in a stochastically resonant manner- which is compatible with experimental observations. The particular strategy employed by the network strongly depends on the level of correlation among the neurons in the network, thus establishing a direct relation between optimal heterogeneity level and spatial correlation.
